# The Care Process Self-Evaluation Tool: a valid and reliable instrument for measuring care process organization of health care teams

**DOI:** 10.1186/1472-6963-13-325

**Published:** 2013-08-19

**Authors:** Deborah Seys, Svin Deneckere, Walter Sermeus, Eva Van Gerven, Massimiliano Panella, Luk Bruyneel, Timothy Mutsvari, Rafaela Camacho Bejarano, Seval Kul, Kris Vanhaecht

**Affiliations:** 1 Department of Public Health, Center for Health Services and Nursing Research, University of Leuven, Kapucijnenvoer 35 4th Floor, Leuven B-3000, Belgium; 2 Department of Public Health, Department of Clinical and Experimental Medicine, Faculty of Medicine, Amedeo Avogadro University of Eastern Piedmont, Novara, Italy; 3 L-Biostat, KU Leuven, Leuven, Belgium; 4 Hasselt Universiteit, Hasselt, Belgium; 5 Department of Nursing, Universidad de Huelva, Huelva, Spain; 6 Department of Biostatistics, Faculty of Medicine, Gaziantep University, Sahinbey, Gaziantep, Turkey; 7 Western Norway Research Network on Integrated Care, Helse Fonna, Haugesund, Norway

**Keywords:** Psychometric properties, Care process, Organization of care, Validity, Reliability, Health care teams, CPSET, Multidisciplinary teams, Multicenter study

## Abstract

**Background:**

Patient safety can be increased by improving the organization of care. A tool that evaluates the actual organization of care, as perceived by multidisciplinary teams, is the Care Process Self-Evaluation Tool (CPSET). CPSET was developed in 2007 and includes 29 items in five subscales: (a) patient-focused organization, (b) coordination of the care process, (c) collaboration with primary care, (d) communication with patients and family, and (e) follow-up of the care process. The goal of the present study was to further evaluate the psychometric properties of the CPSET at the team and hospital levels and to compile a cutoff score table.

**Methods:**

The psychometric properties of the CPSET were assessed in a multicenter study in Belgium and the Netherlands. In total, 3139 team members from 114 hospitals participated. Psychometric properties were evaluated by using confirmatory factor analysis (CFA), Cronbach’s alpha, interclass correlation coefficients (ICCs), Kruskall-Wallis test, and Mann–Whitney test. For the cutoff score table, percentiles were used. Demographic variables were also evaluated.

**Results:**

CFA showed a good model fit: a normed fit index of 0.93, a comparative fit index of 0.94, an adjusted goodness-of-fit index of 0.87, and a root mean square error of approximation of 0.06. Cronbach’s alpha values were between 0.869 and 0.950. The team-level ICCs varied between 0.127 and 0.232 and were higher than those at the hospital level (0.071-0.151). Male team members scored significantly higher than females on 2 of the 5 subscales and on the overall CPSET. There were also significant differences among age groups. Medical doctors scored significantly higher on 4 of the 5 subscales and on the overall CPSET. Coordinators of care processes scored significantly lower on 2 of the 5 subscales and on the overall CPSET. Cutoff scores for all subscales and the overall CPSET were calculated.

**Conclusions:**

The CPSET is a valid and reliable instrument for health care teams to measure the extent care processes are organized. The cutoff table permits teams to compare how they perceive the organization of their care process relative to other teams.

## Background

Recent studies on care quality improvement and patient safety show that health care is still not safe and that the number of adverse events is underestimated [1-3]. Health care across organization levels is often poorly organized, complex, and uncoordinated. Furthermore, not all patients receive consistent, high-quality medical care. The organization of care can be more effective when multidisciplinary teams are involved in its organization and if care is organized around medical conditions and care processes [4,5].

Care processes contain key interventions that support the diagnosis or treatment of patients. These key interventions contain unique bundles of products and services, and temporary firms can effectively achieve their delivery. Temporary firms are health care providers who operate together when a specific well-defined patient group is admitted to a care facility or institution. These teams contain members from different health care professions, have a shared clinical purpose, and have direct care responsibilities [6,7]. They work in a complex environment, under interactive and dynamic conditions, and their membership frequently changes. Therefore, these teams are action teams [7,8]. The challenges for these teams are effective communication, coordination, and control over the care process [7].

A care process has five key characteristics that affect the organization of care: coordination of the care process, patient-focused organization, communication with patients and family, collaboration with primary care, and follow-up of the care process [9]. The Care Process Self-Evaluation Tool (CPSET) assesses these five key characteristics by using a 29-item Likert scale. It is based on the perceptions of team members involved in organizing a care process. The primary study assessing the validity and reliability of this tool was performed in 2007, and statistical analysis of the five factors produced Cronbach’s alpha values between 0.776 and 0.928 [9]. In 2011, an international Delphi study was performed to identify indicators that affect multidisciplinary teamwork in care processes. This study showed that CPSET is a good tool for following up improvements in multidisciplinary teamwork [10].

Since its original validation, CPSET has been used in different organizations and teams. The first aim of this study was to evaluate the stability of the psychometric properties of CPSET. The second aim was to calculate cutoff scores for the subscales and overall score, with the goal of helping health care managers rank the CPSET scores of their teams.

## Methods

### Study design and sample

The present study was a cross-sectional, multicenter study involving 114 organizations in Belgium and the Netherlands. Data were collected between November 2007 and October 2011. The participating organizations were members of the Belgian-Dutch Clinical Pathway Network (http://www.nkp.be) [11-13]. These organizations were offered the opportunity to use the CPSET to evaluate the organization level of care processes after its original validation in 2007. Team leaders decided which team members would complete the CPSET. Teams received feedback on their scores after they sent their data to the central database at Leuven University. We received a total of 3378 questionnaires from team members. Questionnaires with more than three missing values on the 29-item CPSET were excluded from analysis. Thus, 3139 questionnaires were included for secondary analysis in this study. Informed consent was obtained from all participants. The secondary analysis was performed as part of a larger study with the ethical approval of the University Hospitals of Leuven for Belgium (identifiers: B32220096036 and B32330096038) [14].

### Measures

A two-section questionnaire form was used as a data collection instrument. The first part of the questionnaire collected data on demographic characteristics, which included age, gender, profession, and which kind of care process was evaluated. The second part of the questionnaire consisted of the 29-item CPSET. Each item was scored on a 10-point ordered, categorical scale, ranging from totally disagree (1) to totally agree (10) [9]. An average score per subscale (%) and an overall score (%) were calculated.

### Statistical analysis

We performed secondary data analysis on 3139 questionnaires. Descriptive statistics were used to analyze the demographic characteristics. SPSS version 19.0 was used (SPSS Inc., Chicago, IL, USA). Confirmatory factor analysis (CFA) was used to test the structure of the scale. Normed fit index (NFI), comparative fit index (CFI), and adjusted goodness-of-fit index (AGFI) values ≥0.90, and root mean square error of approximation (RMSEA) values < 0.08 were considered to indicate a good fit [15,16]. Cronbach’s alpha analysis was used to measure internal consistency. Cronbach’s alpha values range between 0 and 1, and a high Cronbach’s alpha means that the items are strongly correlated [17]. CFA, NFI, CFI, AGFI, RMSEA, and Cronbach’s alpha were determined using SAS® software (SAS Institute Inc., Cary, NC, USA).

The degree of within-cluster dependence, interclass correlation coefficients (ICCs), and confidence intervals were calculated using a formula derived by Donner and Klar based on analysis of variance [18]. Team- and hospital-level ICCs were calculated to determine to what extent scores at these levels correlate with each other. The Kruskal-Wallis test was used to compare CPSET scores of more than two independent groups. We used this test to compare differences between profession and age and differences between the overall CPSET and its five subscales. The Mann–Whitney test, a test used to assess differences between two independent groups, was used to assess differences between gender, profession, and age of all categories. Kruskal-Wallis tests and Mann–Whitney tests were extracted from StatsDirect 2.7.8 (StatsDirect Ltd, Altrincham, UK) and SPSS version 19.0 (SPSS Inc., Chicago, IL, USA). The level of significance was set to p  < 0.05.

## Results

### Respondents

This study included participants from 92 organizations in Belgium and 22 in the Netherlands. These organizations can be classified as acute hospitals (n = 88), psychiatric hospitals (n = 2), specialized hospitals (n = 9), and primary care organizations (n = 15). Overall, 48.3% of the participants were 40 years or older, 60.31% were female, and more than half (54.76%) were nurses. In total, 283 teams participated. Between one and 28 teams per organization participated. Some teams included a coordinator. Coordinators could be either team leaders or care process coordinators. Some teams included, for example, care logistics in their team; these were classified as others (Table 1).

**Table 1:** Demographic characteristics

**Characteristics**		**N**	**%**
Age (y)			
	20-29	677	21.57
	30-39	870	27.72
	40-49	885	28.19
	50-59	591	18.83
	>60	41	1.30
	Unknown	75	2.39
Gender			
	Male	831	26.47
	Female	1893	60.31
	Unknown	415	13.22
Profession			
	Nurse	1719	54.76
	Medical doctor	543	17.30
	Paramedic	524	16.69
	Coordinator	134	4.27
	Others	219	6.98

### Confirmatory factor analysis

CFA was performed on the original five-factor solution with 29 items [9]. The data of the 3139 participants were used. The four fit indices revealed a good fit for CPSET (AGFI = 0.87; RMSEA = .06; CFI = 0.94; and NFI = 0.93).

### Internal consistency

Internal consistency was measured by calculating Cronbach’s alpha reliability coefficients for each of the five factors. The Cronbach’s alphas for the five factors or subscales were between 0.869 and 0.950 (patient-focused organization, alpha = 0.919; coordination of care, alpha = 0.900; communication with patient and family, alpha = 0.897; collaboration with primary care, alpha = 0.869; follow-up of care, alpha = 0.950).

### Interclass correlation coefficients

We calculated ICCs at team (n = 283) and hospital levels (n = 114). ICCs for each of the five dimensions are shown in Table 2. A team was defined as more than three health care providers who work together around a care process. At the team level, subscale ICCs ranged from 0.127 (collaboration with primary care) to 0.232 (coordination of care). At the hospital level, subscale ICCs ranged from 0.071 (collaboration with primary care) to 0.151 (communication with patient and family). The ICC for the overall CPSET score at the team level was 0.221, whereas the ICC at the hospital level was 0.147. These results showed that there was poor agreement at team and hospital levels and that there was less variation within teams than within hospitals.

**Table 2:** ICCs and 95% confidence intervals

	**Team level**		**Hospital level**	
Subscale scores	ICC	95% CI	ICC	95% CI
Patient-focused organization	0.197	0.150-0.243	0.146	0.085-0.208
Coordination of care	0.232	0.182-0.282	0.131	0.074-0.188
Communication with patient and family	0.210	0.163-0.258	0.151	0.088-0.214
Collaboration with primary care	0.127	0.090-0.164	0.071	0.034-0.108
Follow-up of care	0.215	0.167-0.264	0.134	0.076-0.191
Overall CPSET score	0.221	0.172-0.270	0.147	0.055-0.209

*Abbreviations:ICC* interclass correlation coefficient, *CI* confidence interval.

### CPSET subscales

Gender, age, and profession of team members had a significant impact on the five subscales and the overall CPSET score. Men scored significantly higher on the subscales ‘communication with patient and family’ and ‘collaboration with primary care,’ and on the overall CPSET. Team members younger than 30 years old scored lower on the subscales ‘patient-focused organization,’ ‘communication with patient and family,’ and ‘collaboration with primary care’ and on the overall CPSET. Medical doctors scored significantly higher on the subscales ‘patient-focused organization,’ ‘coordination of care,’ ‘communication with patient and family,’ and ‘collaboration with primary care’ and on the overall CPSET. Paramedics scored significantly higher on the subscale ‘follow-up of care.’ These results are summarized in Table 3.

**Table 3:** Differences in team member demographics according to CPSET subscales

	**Patient-focused organization**	**Coordination of care**	**Communication with patient and family**	**Collaboration with primary care**	**Follow-up of care**	**Overall CPSET score**
	**Average (SD)**	**Average (SD)**	**Average (SD)**	**Average (SD)**	**Average (SD)**	**Average (SD)**
Differences between genders						
Group A: men (n = 831)	74.28 (14.48)	69.80 (13.83)	64.74 (17.35)	68.36 (16.71)	60.86 (18.06)	67.60 (12.71)
Group B: women (n = 1893)	73.58 (13.78)	69.33 (13.42)	61.94 (17.70)	66.11 (15.39)	62.44 (16.56)	66.59 (12.21)
Test			A ≠ B^†^	A ≠ B^†^		A ≠ B^†^
Differences between age groups						
Group A: 20–29 y (n = 677)	72.29 (14.21)	68.69 (13.87)	59.44 (17.86)	65.51 (15.33)	63.64 (15.47)	65.85 (12.73)
Group B: 30–39 y (n = 870)	73.33 (14.03)	63.13 (13.78)	61.37 (18.45)	66.66 (13.35)	61.25 (17.45)	66.31 (12.62)
Group C: 40–49 y (n = 885)	74.13 (14.91)	70.01 (13.61)	63.41 (17.78)	67.58 (16.09)	61.20 (18.05)	67.20 (12.79)
Group D: 50–59 y (n = 591)	75.45 (13.93)	70.54 (14.07)	64.84 (17.06)	67.94 (16.02)	62.64 (16.91)	68.21 (12.34)
Group E: > 60 y (n = 41)	73.93 (14.11)	73.42 (11.54)	67.62 (17.15)	66.50 (19.57)	67.07 (16.26)	69.73 (13.30)
Test	A ≠ B ≠ C ≠ D ≠ E*	A ≠ B ≠ C ≠ D ≠ E*	A ≠ B ≠ C ≠ D ≠ E*	A ≠ B ≠ C ≠ D ≠ E*	A ≠ B ≠ C ≠ D ≠ E *	A ≠ B ≠ C ≠ D ≠ E *
	C > A^†^, D > A^†^, D > B^†^	D > A^†^, E > A^†^, D > B^†^, E > B^†^	B > A^†^, C > A^†^, D > A^†^, E > A^†^, C > B^†^, D > B^†^, E > B^†^	C > A^†^, D > A^†^	A > B^†^, A > C^†^	C > A^†^, D > A^†^, C > B^†^, D > B^†^
Differences between professions						
Group A: medical doctor (n = 543)	76.10 (14.17)	72.64 (13.42)	69.80 (16.34)	72.62 (16.26)	60.76 (18.88)	70.69 (12.35)
Group B: nurses (n = 1719)	73.03 (14.80)	69.17 (13.84)	58.80 (18.00)	64.83 (16.00)	61.97 (16.70)	65.49 (12.83)
Group C: coordinator of care process (n = 134)	73.60 (11.7)	65.17 (12.17)	61.09 (14.73)	65.51 (12.52)	56.45 (16.27)	64.24 (9.63)
Group D: paramedic (n = 524)	72.89 (13.66)	68.82 (14.19)	64.50 (17.72)	67.69 (15.38)	64.88 (16.42)	67.74 (12.57)
Test	A ≠ B ≠ C ≠ D*	A ≠ B ≠ C ≠ D*	A ≠ B ≠ C ≠ D*	A ≠ B ≠ C ≠ D*	A ≠ B ≠ C ≠ D*	A ≠ B ≠ C ≠ D*
	A > B^†^, A > C^†^, A > D^†^	A > B^†^, A > C^†^, A > D^†^, B > C^†^, D > C^†^	A > B^†^, A > C^†^, A > D^†^, D > C^†^, D > B^†^	A > B^†^, A > C^†^, A > D^†^, D > C^†^	D > A^†^, A > C^†^, D > B^†^, B > C^†^, D > C^†^	A > B^†^, A > C^†^, A > D^†^, D > B^†^, D > C^†^

*Abbreviations:SD* standard deviation.

*Kruskal Wallis Test, P  < 0.05.

^†^Mann–Whitney test, P  < 0.05.

### CPSET cutoff scores

On the basis of the individual scores of the 3139 participating health care providers, we calculated cutoff scores for the five subscales and the overall CPSET (Table 4). Percentiles were used in the cutoff table based on a normal distribution of the subscale scores. The subscales ‘communication with patient and family’ and ‘follow-up of care’ had a broader range of scores than the other subscales and the overall CPSET.

**Table 4:** Cutoff table

**Percentile**	**Patient-focused organization**	**Coordination of care**	**Communication with patient and family**	**Collaboration with primary care**	**Follow-up of care**	**Overall CPSET score**
P10	55.00	51.43	37.50	46.67	38.75	50.57
P20	63.33	60.00	47.50	53.33	48.75	57.00
P30	68.33	64.29	55.00	60.00	55.00	61.46
P40	71.67	67.14	60.00	63.33	60.00	64.68
P50	75.00	71.43	65	70.00	63.75	67.79
P60	78.33	74.29	67.50	70.00	68.75	70.54
P70	80.00	77.14	72.50	76.67	71.25	73.52
P80	85.00	81.43	77.50	80.00	76.25	76.90
P90	90.00	85.71	85.00	86.67	81.25	82.41

## Discussion

This paper describes the psychometric properties of the CPSET and defines CPSET cutoff scores. The psychometric properties of the CPSET showed that this tool is valid and reliable for evaluating the organization of a care process as perceived by team members. The original five-factor structure with 29 items was confirmed by CFA. The reliability of the CPSET was measured by Cronbach’s alpha. The Cronbach’s alpha results in this study varied between 0.869 and 0.950 and were higher than those reported in the original 2007 study of Vanhaecht et al. (Cronbach’s alpha = 0.776-0.928) [9]. This indicates that the scale is still reliable.

A multilevel analysis was performed at team and hospital levels. Our results showed that the ICCs of scores at the team level were higher than those at the hospital level. This means that there was less variance in CPSET scores within teams than within hospitals, which was expected. The ICCs in our study were low, and most of the variation could be explained by team and hospital variations. One possible reason for the low ICCs in our study was that teams were composed of professionals from different disciplines, with each team member having different perceptions about the organization of care. As shown in Table 3, profession, age, and gender significantly influenced CPSET scores. Medical doctors scored significantly higher than other health professionals on the overall CPSET scale and on the following subscales: ‘patient focused organization,’ ‘coordination of care,’ ‘communication with patient and family,’ and ‘collaboration with primary care.’ Paramedics scored significantly higher on the subscale ‘follow-up of care.’ Men scored significantly higher on the overall CPSET scale and the subscales ‘communication with patient and family’ and ‘collaboration with primary care.’ Team members between 20 and 39 years old scored significantly lower on the subscale ‘communication with patient and family’ than those in other age categories. Significant differences were found between younger ( < 40 years) and older (> 50 years) health care professionals on the subscale ‘coordination of care.’ Coordinators of care pathways scored lower on the subscales ‘coordination of care’ and ‘follow-up of care,’ perhaps because they tend to be more critical of the organization of care.

The differences we observed in the perceptions of medical doctors and nurses are consistent with those observed in previous research. In the present study, physicians perceived teams to be more organized than nurses in terms of teamwork, collaboration, and communication with nurses, which is consistent with the finding that physicians generally perceive teamwork to be better coordinated [19-21]. A negative correlation exists between professional autonomy and the level of nurse-physicians collaboration [22]. Different perspectives in communication can be caused by hierarchical factors, gender, different patient care responsibilities, different perceptions of requisite communication standards, and differences in training methods for nurses and doctors [19]. Communication skills training can improve patient-nurse communication but not patient-doctor communication. Skills training that contains patient-centered communication can increase information exchange and continuity of care for patients [23].

The lowest CPSET scores were observed on the subscale ‘communication with patient and family’ and ‘follow-up of care.’ Organizations need to improve on priorities, communication, and coordination of care, as suggested by Bates et al. [24]. Relational coordination can be used to improve ‘follow-up of care.’ This framework can lead to better quality of care for patients, and health care providers reported fewer adverse events [25]. Effective and safe hospital care depends on good teamwork. Greater teamwork results in higher patient satisfaction rates, higher nurse retention, and lower hospital costs [26]. Multidisciplinary teamwork is essential for quality health care.

The 2012 review of Deneckere et al. showed that multidisciplinary teamwork can be supported by using care pathways [7]. Care pathways can improve the work environment and organization of care, and can have a positive impact on the well-being of health care providers [7,27,28]. Further research is needed on using the CPSET to study the effect of coordinating mechanisms, such as care pathways, on how health care providers perceive the organization of care.

Although the CPSET has been used for several years, health care managers have problems interpreting the CPSET scores of their team members. Therefore, we compiled a table of cutoff scores that will permit health care managers and team members to compare how they perceive the organization of their care process relative to other team members. By using the cutoff table as a starting point, team members can discuss how they can improve the organization of care. When different teams of the same care facility or institution complete the CPSET, the cutoff scores will help health care managers rank the teams in that facility or institution. However, this should be done carefully. The primary aim of the cutoff scores is to initiate discussions within teams. For example, they can look for possible reasons why they perceive the organization of care to be low and what they expect from other team members. They can also learn from actions taken by other teams. We hypothesize that teams that use care bundles, care pathways, or evidence-based protocols will have CPSET scores in the higher percentiles compared with teams that do not use quality improvement initiatives. But further research is needed to determine whether the structured care associated with quality improvement initiatives does indeed change the perception of health care providers according to the actual organization of care.

Some limitations of our study should be considered. One limitation is the risk of social desirability and selection bias. Team leaders and coordinators of care processes decided which specific team members would complete the CPSET. Hence, it is possible that not all team members or health care professionals involved in a specific care process were surveyed. Another concern is that the results of this study are based on data from two countries: Belgium and the Netherlands. Therefore, a comparable study should be conducted in additional countries. The validity of the CPSET is currently being tested in French, Norwegian, Italian, Portuguese, English, and German languages.

## Conclusions

The CPSET is a valid and reliable tool for measuring the organization of care as perceived by involved health care providers. Some of the CPSET scores depend on age, gender, and profession. Team leaders can use the CPSET to evaluate how their team members perceive the organization of care. The cutoff scores presented in this study will aid health care managers rank their team, identify differences between teams within a care facility or institution, and analyze the needs of teams in their collaborative search for excellence.

## Competing interests

The authors declare that they have no competing interests.

## Authors’ contributions

KV, WS, and MP defined the design of the study and organized the data collection. SD, EVG, and DS coordinated the data cleaning and supported the participating organizations. LB, TM, RCB, and SK performed the statistical analysis. KV, SD, and DS prepared the first draft of the manuscript. All authors discussed the results and approved the final version of the manuscript.

## Pre-publication history

The pre-publication history for this paper can be accessed here:

http://www.biomedcentral.com/1472-6963/13/325/prepub
